# Aging and End Stage Renal Disease Cause A Decrease in Absolute Circulating Lymphocyte Counts with A Shift to A Memory Profile and Diverge in Treg Population

**DOI:** 10.14336/AD.2018.0318

**Published:** 2019-02-01

**Authors:** Geraldo Rubens Ramos Freitas, Maria da Luz Fernandes, Fabiana Agena, Omar Jaluul, Sérgio Colenci Silva, Francine Brambate Carvalhinho Lemos, Verônica Coelho, David-Neto Elias, Nelson Zocoler Galante

**Affiliations:** ^1^Division of Nephrology, and; ^2^Renal Transplant Service, Hospital das Clinicas, University of Sao Paulo School of Medicine, Sao Paulo, Brazil.; ^3^Division of Geriatrics, Hospital das Clinicas, University of Sao Paulo School of Medicine, Sao Paulo, Brazil.; ^4^Laboratory of Immunology, Heart Institute, University of Sao Paulo School of Medicine. Institute for Investigation in Immunology, Sao Paulo, Brazil.

**Keywords:** T lymphocytes, B lymphocytes, Chronic Kidney Disease, Kidney transplantation, Immune senescence

## Abstract

There is a growing number of elderly kidney transplant (Ktx) recipients. Elderly recipients present lower acute rejection rates but higher incidence of infection and malignancies. Aging *per se* seems to result in a shift to memory profile and chronic kidney disease (CKD) in premature immunological aging. Understanding aging and CKD effects on the immune system can improve elderly Ktx immunosuppression. We analyzed the effects of aging and CKD in the immune system, comparing healthy adults (HAd) (n=14, 26±2y), healthy elderly (HEld) (n=15, 79±7y), end stage renal disease (ESRD) adults (EnAd) (n=18, 36±7y) and ESRD elderly (EnEld) (n=31, 65±3y) prior to Ktx regarding their naïve, memory and regulatory T and B peripheral lymphocytes. Aging and ESRD presented additive effect decreasing absolute numbers of B and T-lymphocytes, affecting memory, naive and regulatory subsets without synergic effect. Both resulted in higher percentages of T memory subsets and opposing effects on regulatory T (TREG) subsets, higher percentage in aging and lower in ESRD. Combined effect of aging and ESRD also resulted in higher regulatory B cell percentages. In addition to global lymphopenia and TCD4^+^ memory shift in both aging and ESRD, aging shifts to an immunoregulatory profile, inducing a increase in TREG percentages, contrasting with ESRD that decreases TREGs. Differential immunosuppression regimens for elderly Ktx may be required. (ClinicalTrials.gov number: NTC01631058).

A challenge in kidney transplantation is to improve the short- and long-term outcomes for elderly recipients. The rates of infection [1] and malignancy [2] after transplantation are significantly higher among elderly recipients and acute rejection episodes are less frequently reported [3].

Although these events are often attributed to over-immunosuppression with lower clearance of drugs [4, 5], aging influences many aspects of the immune system, including lymphocyte counts, subset differentiation and function.

Studies with healthy volunteers have shown that aging is followed by a progressive T-cell lymphopenia in peripheral blood, with accumulation of memory T-lymphocytes in contrast to naive T-cells [6, 7]. T-cells with cell surface expression of regulatory markers have also shown to be more frequent among healthy elderly [8].

Information about the effect of aging on B-lymphocytes is conflicting. In most studies B-cell lymphopenia is a common finding among healthy elderly [9, 10], but both age-related increase [10, 11] and decrease [12, 13] in B-cells with memory phenotype have been reported. Little is available about the effects of aging among B-cells with expression of regulatory markers.

End stage renal disease (ESRD) also induces phenotype and functional changes in peripheral lymphocytes. Although most studies agree that a T-cell lymphopenia accompanies the progressive loss of renal function [14-16], the numbers of naive T-cells have been reported to be reduced [17, 18] or unchanged [16] among ESRD. There is also no agreement regarding the frequencies of memory T-cells among uremic patients. Chung et al. reported that central memory and effector memory T-lymphocytes accumulate in peripheral blood of uremic patients as compared to healthy controls [18], whereas Yoon et al. showed a significant reduction of the same populations among uremic patients [17]. Also, the numbers of regulatory T-cells have been reported to be decreased compared to healthy controls, but with unchanged relative percentages [15]. Also, a decrease in naive B-cell population has been reported [19], as well as a reduction in the percentage of memory and regulatory B-cells in uremic patient [20].

Recently lymphocyte profiles have been associated with allograft outcomes. Memory subsets of T cells have been related to acute rejection occurrence and resistance to tolerance induction in graft recipients [21, 22]. Among TCD4^+^ memory lymphocytes, subpopulations can be predominantly found in lymphoid tissue (T central memory) or in non-lymphoid tissue (T effector memory), but their specific roles modifying allograft outcomes is still poor defined. Greater interstitial infiltration of B cells with CD27^+^ (memory) and CD38^+^ expression are commonly found in acute cellular rejection of kidney allografts and correlate with lower steroid response [23]. The higher occurrence of regulatory T cells (CD4^+^CD25^+^FoxP3^+^) correlates with stable graft function and immune tolerance occurrence [24], greater glomerular filtration rates and lower acute rejection incidence [25]. Also, relative higher regulatory B cells (CD19^+^CD24^Hi^CD38^Hi^) occurrence relates to stable graft function [26] and operational tolerance [27].

Elderly recipients present different clinical outcomes compared to younger counterparts and both, ESRD and aging may change lymphocyte subsets profile. Characterizing the lymphocyte subset changes in ESRD-aging co-occurrence would help to understand these clinical discrepancies.

Lymphocyte subset characterization, patient selection, degree of renal dysfunction and disease definition among available studies is quite variable, making comparisons difficult. Also, the effects of aging or chronic kidney disease (CKD) were assessed separately in different studies and no studies analyzed the T- and B-cell compartment simultaneously.

In this study, we analyzed, all together, the effects of aging and ESRD in the subsets of peripheral blood T and B lymphocytes. The comprehension of these variables effects on lymphocyte profile may be determinant to immunosuppression individualization of elderly kidney transplant recipients.

## MATERIAL AND METHODS

### Study design and population

This is a cross-sectional study to investigate aging and ESRD related quantitative changes in peripheral blood T- and B-cell maturation stages comparing healthy subjects (healthy elderly and healthy younger adults) with ESRD patients (elderly and younger adults).

Healthy subjects and ESRD patients were enrolled at our hospital from September 2012 to July 2015. Fourteen healthy adults (>18 and ≤45years) (HAd) and 15 healthy elderly (>60 years) (HEld) participated in this study. Self-reports of health were used to assess the health condition of HAd. All HEld are volunteers regularly followed at the outpatient unit of the Geriatric Division of our institution and free of chronic illness, such as systemic arterial hypertension (SAH), diabetes mellitus (DM), malignancy or psychiatric disorders. All healthy subjects also did not present chronic viral infections or had presented acute illness or been vaccinated for at least 60 days. Healthy subjects were free of any medication for at least 6 months.

Forty-nine patients with ESRD, already on dialysis, were recruited at hospital admission for their first kidney transplantation. Eighteen were younger ESRD adult patients (EnAd), and 31 were ESRD elderly patients (EnEld). The elderly recipients were those included in the nEverOld study [28] (Clinical Trials identifier: NTC01631058).

The study was approved by institutional board of ethics in research. All individuals provided informed consent prior to enrollment.

### Blood collection and cell preparation

A sample of 20mL of heparinized whole blood was collected from healthy subjects at study recruitment, and immediately prior to kidney transplantation from ESRD patients. Total leukocyte and lymphocyte counts were performed according to standard methods. Peripheral blood mononuclear cells (PBMC) were separated by Ficoll density gradient centrifugation and cryopreserved for further analysis.

**Figure 1.**
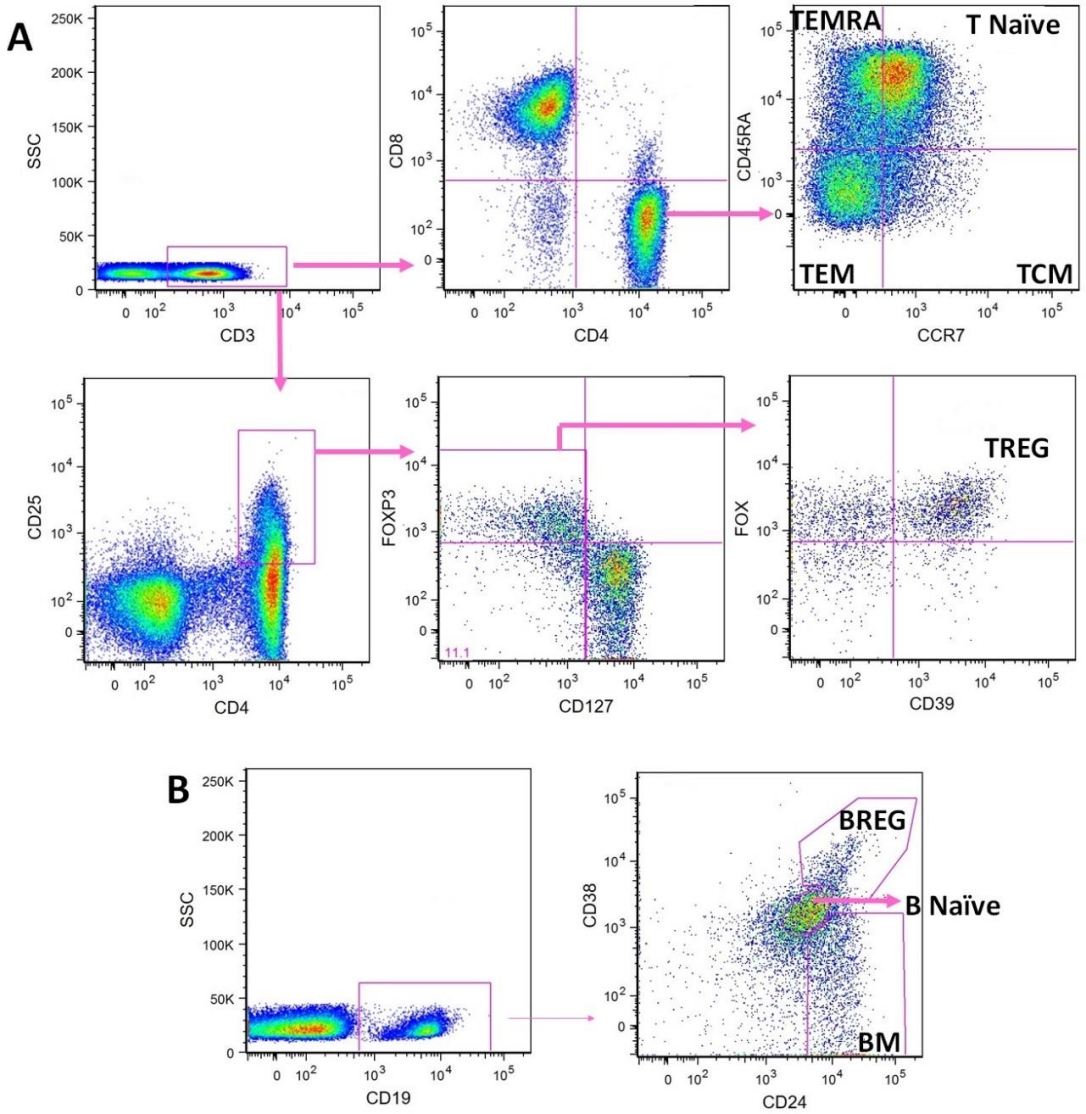
**Flow cytometry characterization of peripheral blood T and B cell subsets**. Fluorescence minus one (FMO) controls were set up for CD127, FoxP3, CD39, CCR7 (CD197) and CD45RA. Panel A represents the strategy for T cell subsets characterization and panel B for B cell subsets characterization. TCM - T central memory, TEM - T effector memory, TEMRA - T effector memory with RA reexpression, TREG - regulatory T cells, BM - B memory, BREG - regulatory B cells.

### Flow cytometry identification of T- and B-lymphocytes subpopulations

PBMC were thawed, washed, and then stained with titrated mouse anti-human monoclonal antibodies. Anti-CD4-fluorescein isothiocyanate (FITC) (OKT4), anti-CD19-FITC (HIB19), anti-CCR7 (CD197)-Phycoerythrin (PE) (3D12), anti-CD45RA-PE-Cy7 (HI100), anti-CD25-PE (BC96), anti-CD127-PE-Cy7 (RDR5), anti-FoxP3-Peridinin Chlorophyll Protein Complex (PerCp)-Cy5.5 (PCH101) and anti-CD39- allophycocyanin (APC) (A1) antibodies were from eBiosciences (San Diego, CA, USA). Anti-CD3-APC-Cy7 (SK7), anti-CD8-AmCyan (SK1), anti-CD24-PE-Cy7 (ML5), anti-CD38-PerCp-Cy5.5 (HIT2) and BD Multitest™ CD3/CD8/CD45/CD4 [anti-CD3-FITC (SK7), anti-CD8-PE (SK1), anti-CD45-PerCP (2D1 (HLe-1)) and anti-CD4-APC (SK3)] were from BD Biosciences (Heidelberg, Germany). For intra-cellular staining of FOXP3, cells were washed, fixed and permeabilized with Foxp3 staining buffer from eBioscience (San Diego, CA, USA) immediately after surface-staining. At least 0.5 × 10^5^ events in the lymphocyte region were acquired. Fluorescence minus one (FMO) controls were set up for CD127, FoxP3, CD39, CCR7 (CD197) and CD45RA markers.

**Table 1:** Demographic data.

	HAd (n=14)	HEld (n=15)	EnAd (n=18)	EnEld (n=31)	P
Age (years)	26±2	79±7^a^	36±7^b^,^d^	65±3^c^,^e^,^f^	<0.001
Gender (Male) n (%)	5 (36)	1 (7)	7 (39)^d^	18 (58)^e^	0.01
BMI (Kg/m^2^)	---	---	24(22.4-25.6)	25(22.8-27.2)	0.206#
**Comorbidities**					
SAH n (%)	0	0	16 (89)^b^,^d^	31 (100)^e^	<0.001
Diabetes mellitus n (%)	0	0	3 (17)	15 (48)^c^,^e^	<0.001
Glomerulopathy n (%)	0	0	3 (17)	2 (7)	0.107
ADPKD n (%)	0	0	1 (6)	2 (7)	0.41
**Renal replacement data**					
Time on RRT (months)	---	---	32 (14-45.5)	36 (21-54)	0.23#
RRT (HD) n (%)	---	---	17 (94)	28 (90)	1.0#
**Laboratorial data**					
Albumin	---	---	4.4(4.17-4.62)	4.3(3.6-4.7)	0.4#
Hemoglobin	---	---	12.2(9.9-13.1)	12.6(11-12.9)	0.88#
PTH	---	---	236(80.5-608)	237(130-574)	0.868#

HAd - healthy adults; HEld - healthy elderly; EnAd - end stage renal disease adult patients; EnEld - end stage renal disease elderly patients, BMI - body mass index, SAH - systemic arterial hypertension, ADPKD - Autosomal dominant polycystic kidney disease, RRT - renal replacement therapy, HD - hemodialysis, PTH - parathyroid hormone.

# Comparison of EnAd and EnEld only

a Significant difference between: ^a^HAd and HEld;

b HAd and EnAd;

c HAd and EnEld;

d HEld and EnAd;

e HEld and EnEld;

f EnAd and EnEld.

Flow cytometry was performed in FACSCanto-II (BD Biosciences) cytometer. We used FlowJo 9.1 software (TreeStar Inc, San Carlos, CA, USA) for analysis. After exclusion of cell doublets and debris, sequential gating of PBMC was performed in the lymphocyte region. The gating strategies used to define T- and B-cell subsets are shown in Figure 1. The lymphocyte evaluated were T (CD45^+^CD3^+^), TCD8 (CD45^+^CD3^+^CD8^+^), TCD4 (CD45^+^CD3^+^CD4^+^), B (CD19^+^) and the subpopulations TCD4 naive (CD3^+^ CD4^+^CCR7^+^CD45RA^+^) (T naive), TCD4 central memory (TCM) (CD3^+^CD4^+^CCR7^+^CD45RA^-^), TCD4 effector memory (TEM) (CD3^+^CD4^+^CCR7^-^CD45RA^-^), TCD4 effector memory with RA reexpression (TEMRA) (CD3^+^CD4^+^CCR7^-^CD45RA^+^), regulatory TCD4 (TREG) (CD3^+^CD4^+^CD25^hi^CD127^-^FoxP3^+^CD39^+^), B naive (CD19^+^CD24^int^CD38^int^), B memory (BM) (CD19^+^CD24^+^CD38^-^) and regulatory B cells (BREG) (CD19^+^CD24^hi^CD38^hi^).

Lymphocyte subsets absolute counts were calculated using the percentages obtained in flow cytometry and the lymphocyte counts achieved in standard method blood counts on fresh blood before PBMC separation. The subset percentages analyzed were referred to total lymphocyte counts for T, TCD4, TCD8 and B cells, to TCD4 cells for T naïve, TCM, TEM and TEMRA, to CD4^+^CD25^+^ cells for TREG and to B cells for B naive, BM and BREG.

### Statistical analysis

Kolmogorov-Smirnov or Shapiro-Wilk tests were used to test for normal distribution of continuous variables. The non-normally distributed variables underwent log-transformation to normalize the distribution, and the log-transformed data were compared when appropriate. Student’s t-test was used to compare normally distributed continuous variables and Mann-Whitney U-test was used for non-normally distributed variables. The Chi-square or Fisher’s exact test were used to compare nominal variables. Two-way analysis of variance (ANOVA) was used to test for aging vs ESRD interactions. Data are presented as medians and interquartile ranges [median (p25-p75)]. A p value <0.05 was considered significant. To evaluate aging effects HAd was compared with HEld and EnAd with EnEld. To evaluate ESRD effects HAd was compared with EnAd and HEld with EnEld.

All analyses were performed with SPSS-20 (IBM-Corp., Armonk, NY, USA) and GraphPad Prism 6 software (GraphPad Software Inc., La Jolla, CA, USA).

## RESULTS

### Study population

As expected median age was significantly lower for younger adults compared to elderly for both healthy volunteers [26±2 vs. 79±7, p<0.001] and ESRD patients [36±7 vs. 65±3, p<0.001]. HEld were older than EnEld, and EnAd were older than HAd. There was a higher prevalence of female in HEld. As expected, ESRD groups presented a high prevalence of SAH and DM. EnAd and EnEld did not differ in the prevalence of SAH, DM, glomerulopathy and autosomal dominant polycystic kidney disease (Table 1). All patients presented glomerular filtration rate lower than 15 ml/min/1,73 m^2^ and were all on dialysis. ESRD groups presented similar time on renal replacement therapy, hemodialysis prevalence, body mass index, albumin, hemoglobin and parathyroid hormone levels. Specific dialysis data such as Kt/V, dialysis session duration or erythropoietin exposure were not available.

### The effects of aging in lymphocyte subpopulations

#### Healthy Individuals

**Figure 2.**
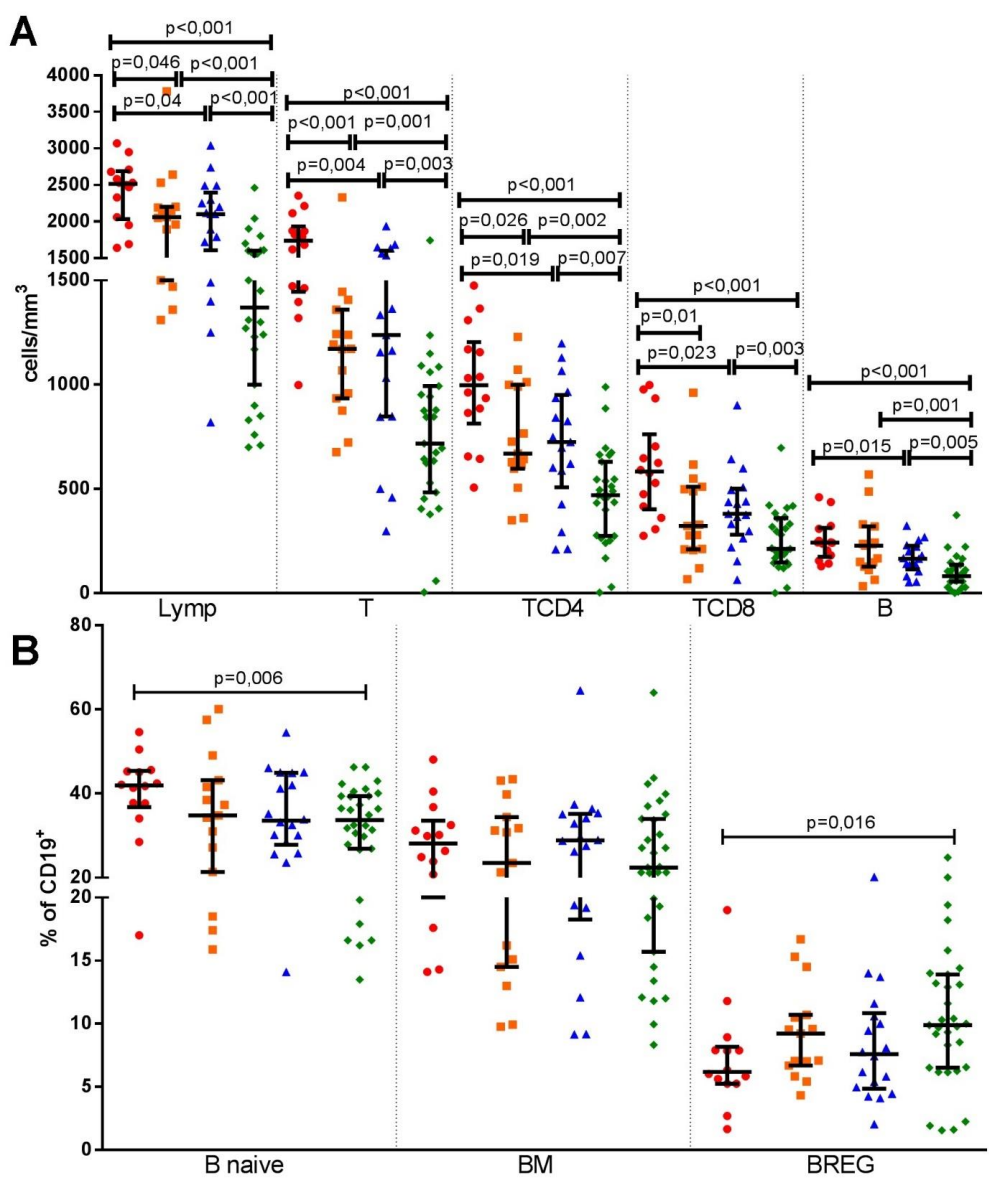
**Aging and end stage renal disease effects in absolute total lymphocytes, T-, TCD4-, TCD8- and B- cells (A) and in B-cells subsets percentages (B)**. Healthy adults (n=14) ( 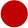), healthy elderly (n=15) ( 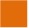), end stage renal disease adult patients (n=18) ( 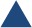) and end stage renal disease elderly patients (n=31) ( 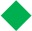) absolute counts and percentages are shown in the same repeatedly order in each cell subset analysis. Lymp - total lymphocyte, T - T lymphocyte, TCD4 - T helper, TCD8 - T cytotoxic, B - B lymphocyte, BM - B memory, BREG -regulatory B cells. Bars represent median and interquartile ranges.

**Figure 3.**
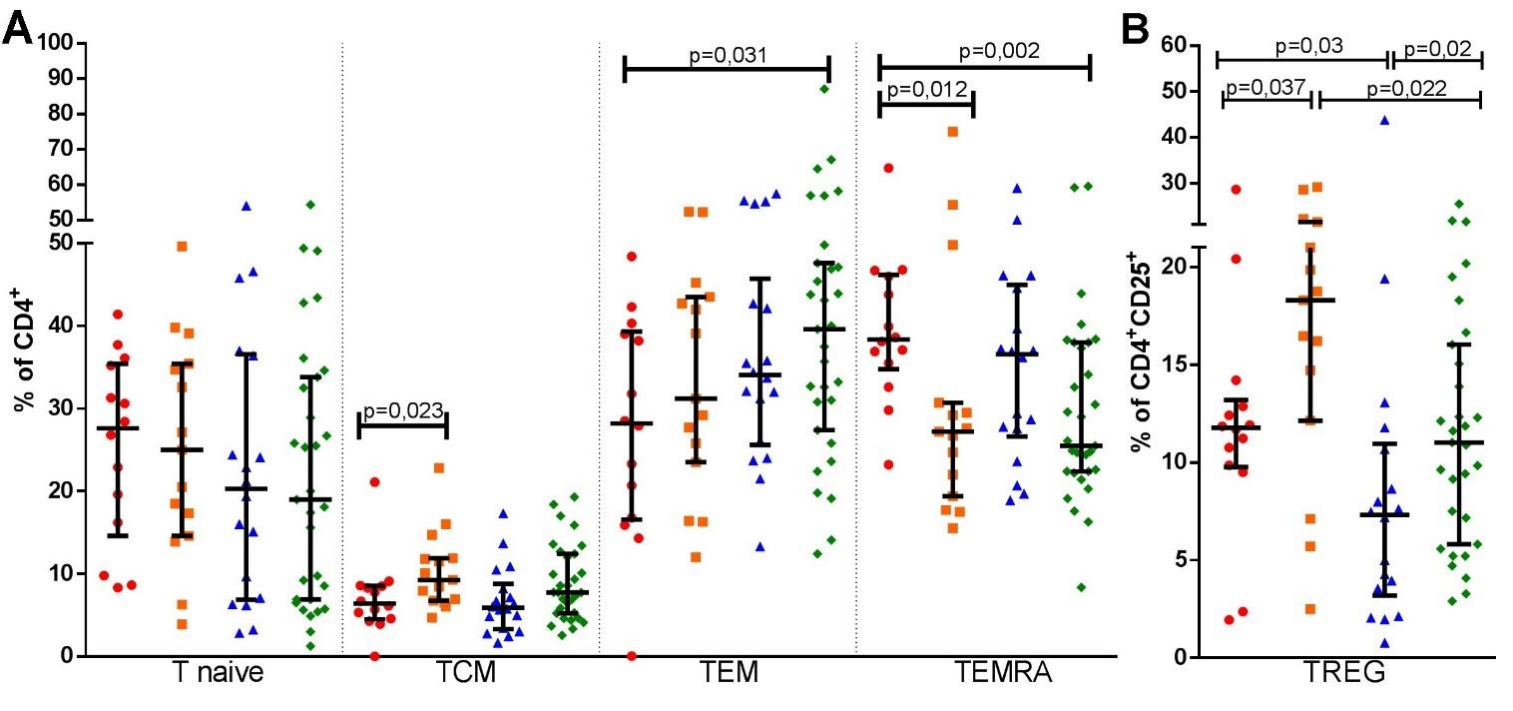
**Aging and end stage renal disease effects in T - cells subsets percentages, T naïve, TCM, TEM, TEMRA (A) and TREG (B)**. Healthy adults (n=14) ( 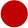), healthy elderly (n=15) ( 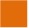), end stage renal disease adult patients (n=18) ( 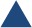) and end stage renal disease elderly patients (n=31) ( 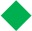) subsets percentages are shown in the same repeatedly order in each cell subset analysis. TCM - T central memory, TEM - T effector memory, TEMRA - T effector memory with RA reexpression, TREG - regulatory T cells. Bars represent median and interquartile ranges.

The effect of aging in healthy subjects were determined with comparison of HEld with HAd. HEld presented lower absolute counts of total lymphocytes, T, TCD4 and TCD8 lymphocytes (Fig. 2A), as well as lower absolute count (Table 2 and supplementary Fig. 2A) and percentage of TEMRA cells (Fig. 3A). Although the absolute counts of TCM and TREG cells (Table 2 and supplementary Fig. 2A and 2B) did not significantly differ, higher percentages of both populations were observed in the HEld (Fig. 3A and 3B). There were no significant differences among the B-cell subsets (Fig. 2B).

#### ESRD individuals

To determine the effect of aging on lymphocyte subpopulations of ESRD patients, we compared EnEld with EnAd. The aging effect in ESRD resembled those in healthy subjects. EnEld presented significant lower absolute counts of total lymphocytes, T, TCD4, TCD8 and B-cells (Fig. 2A), as well as lower absolute counts of TEM and TEMRA (table 2 and supplementary Fig. 2A). The absolute counts of TREG did not significantly differ (table 2 and supplementary Fig. 2B), but the percentages of cells with this phenotype were higher in the CD4^+^CD25^+^ population of EnEld (Fig. 3B). Significant lower absolute counts of B naive and BM subsets were also observed in EnEld (Table 2 and supplementary Fig. 1B). There were no significant differences in percentages of the B-cell subsets (Fig. 2B).

### The effect of ESRD in lymphocyte subpopulations

Next, we determined the effect of ESRD in lymphocyte subpopulations of adults comparing ESRD patients with healthy subjects. The results of ESRD were very similar to those of aging.

#### Adult individuals

We first compared EnAd with HAd. We found that EnAd presented lower absolute counts of total, T, TCD4, TCD8 and B lymphocytes (Fig. 2A and Table 2). Among TCD4 cell subsets, EnAd presented lower absolute counts of T naive and TEMRA cells (Table 2 and supplementary Fig. 2A). Also, the absolute counts of TREG were not significantly different between groups (table 2 and supplementary Fig. 2B) but the percentage of cells with this phenotype was lower among EnAd (Fig. 3B). EnAd also presented lower absolute numbers of B naive and BM cells (Table 2 and supplementary Fig. 1B), but no significant differences among percentages of B-cell subsets (Fig. 2B).

#### Elderly individuals

To evaluate the effect of ESRD in lymphocyte subpopulations in the elderly cohorts, we compared EnEld with HEld subjects. EnEld presented lower absolute counts of total lymphocytes, T, TCD4 and B lymphocytes (Fig. 2A and Table 2), with lower B-cell percentage (supplementary Fig. 1A). Among TCD4, the absolute counts of T naive, TCM, TEMRA and TREG lymphocytes subsets were also lower (table 2 and supplementary Fig. 2A and 2B). EnEld presented a lower percentage of TREG lymphocyte compounds (Fig. 3B). Significant lower absolute counts of B naive, BM and BREG subsets (Table 2 and supplementary Fig. 1B) were also observed with no percentage differences (Fig. 2B).

### The interaction of aging and ESRD in lymphocyte subpopulations

We further investigated the combined effect of aging and ESRD in lymphocyte subpopulations comparing EnEld with HAd. EnEld presented lower absolute counts of total lymphocytes and all lymphocyte subpopulations, namely T, B, TCD4, TCD8, T naive, TCM, TEM, TEMRA, TREG, B naive, BM and BREG cells (Fig. 2A, supplementary Fig. 1B, 2A, 2B and Table 2).

EnEld also presented lower percentages of T, TCD8, TEMRA, B and B naive lymphocytes (Fig. 2B, Fig. 3A and supplementary 1A). Despite the general lower absolute counts in lymphocyte subpopulations, EnEld presented higher percentage of TEM and BREG lymphocytes (Fig. 2B and 3A). TREG percentages did not differ comparing EnEld with HAd (Fig. 3B).

**Table 2:** Effects of aging and end stage renal disease in the absolute numbers (cells/mm^3^) of lymphocyte subpopulations among healthy adults, healthy elderly, adult ESRD patients and elderly ESRD patients.

	HAd (n=14)	HEld (n=15)	EnAd (n=17)	EnEld (n=27)
Total Lymp	2515 (2033-2688)	2060 (1500-2200)^a^	2100 (1605-2395)^b^	1370 (1000-1600)^c^,^e^,^f^
T	1737 (1446-1933)	1172 (934.3-1360)^a^	1238 (847.3-1599)^b^	718 (483.7-992.8)^c^,^e^,^f^
TCD4	997.2 (813.1-1205)	670 (597.2-999.3)^a^	724.9 (508-951)^b^	470.2 (274.5-631.2)^c^,^e^,^f^
TCD8	584 (403.2-761.8)	322.5 (210.1-510.6)^a^	380.5 (281.1-501.3)^b^	213 (147.9-359.8)^c^,^f^
B	243 (175.2-313.1)	229 (127.7-321.4)	165.1 (115.5-227.9)^b^	82 (56.5-137.2)^c^,^e^,^f^
T naive	228.5 (111.1-337.4)	155.3 (86.2-246.9)	106.1 (45.4-234.2)^b^	89 (25.1-138)^c^,^e^
TCM	66.9 (44.4-86.8)	68.4 (39.6-85.6)	59.1 (16.5-75.6)	35 (19.6-62)^c^,^e^
TEM	262.5 (156.1-421.4)	201.2 (145.8-254.2)	234.8 (162.3-338.8)	164.4 (88.6-230.5)^c^,^f^
TEMRA	354.1 (263.8-470.3)	190.5 (104.3-315)^a^	293.4 (107.4-323.3)^b^	120 (64.6-179.2)^c^,^e^,^f^
TREG	24 (10.6-29.2)	24.1 (15.4-34.5)	12.9 (3 -16.5)^d^	8.9 (4.8-18.1)^c^,^e^
B naive	106.5 (71.6-132.5)	87.4 (34.7-116.2)	49.7 (33-75.9)^b^	23.4 (11.2-47.7)^c^,^e^,^f^
BM	69.1 (42.6-85.1)	43.9 (23.9-70.8)	45.4 (26.5-52.5)^b^	26.4 (7-32)^c^,^e^,^f^
BREG	15.5 (8.9-22.3)	18.5 (9.8-30.5)	12.3 (7.1-17.3)	7.5 (2.4-15.7)^c^,^e^

HAd - healthy adults; HEld - healthy elderly; EnAd - end stage renal disease adult patients; EnEld - end stage renal disease elderly patients; Lymp - lymphocyte; T - T lymphocyte; TCD4 - T helper; TCD8 - T cytotoxic; B - B lymphocyte; TCM - T central memory; TEM - T effector memory; TEMRA - T effector memory with RA reexpression; TREG - regulatory T cells; BM - B memory; BREG - regulatory B cells. Significant difference between:

a HAd and HEld;

b HAd and EnAd;

c HAd and EnEld;

d HEld and EnAd;

e HEld and EnEld;

f EnAd and EnEld

To strengthen the analysis of the combined effects of aging and ESRD testing their possible interaction, we performed a two-way ANOVA with 4 groups in a two-by-two factorial design. The independent variables were age (elderly vs. younger adults) and ESRD (vs. healthy volunteers).

We attributed the lymphocyte subset change to ESRD if both grouped comparisons (HAd vs. EnAd and HEd vs. EnEld) were in agreement. Similarly, we attributed the lymphocyte subset change to aging if both grouped comparisons (HAd vs. HEd and EnAd vs. EnEld) converged. Isolated ANOVA differences for each condition were also considered relevant.

All differences found in the previous analysis attributed to ESRD and aging were confirmed. There was no synergetic effect in absolute counts or percentages of lymphocyte subsets (table 3). In this analysis, again both conditions influenced the percentage of TREG, but with opposite effects. aging resulted in higher percentage of TREG whereas ESRD in lower percentage of TREG.

The following differences were found in two-way ANOVA but not described in the previous comparison. Elderly individuals had significant lower T naive and BM absolute counts with lower percentage of TCD8 and TEMRA and higher percentage of TCM lymphocytes than younger adults. ESRD patients had lower absolute counts of T naive, TCM, BM and BREG with lower percentages of B-cells and higher percentage of TEM cells than healthy volunteers (Table 3).

## DISCUSSION

In this study, we analyzed circulating lymphocyte subsets of elderly and young adults with ESRD on admission for their kidney transplantation, along with healthy volunteers, to test the hypothesis that aging and ESRD quantitatively modify peripheral blood T- and B- cell maturation and differentiation profiles. We found that aging affects lymphocyte subpopulation profile of ESRD patients in a similar pattern it affects individuals without ESRD. Aging was associated with global reduction in the absolute numbers of total lymphocytes, TCD4 and TCD8 subsets. The subpopulation changes were more intense in the TCD4 subsets with lower T naive and TEMRA absolute counts and higher TCM percentage. We found a higher percentage of TREGs without changes in TREG absolute counts. For the B- cells, aging resulted in lower absolute BM counts, with similar subsets percentages.

Our findings on the T- cells are in agreement with others who described elderly individuals with lower T naive lymphocyte population [6-8, 29], higher TCM [7] and higher TREG percentage [8, 29, 30]. However, our data conflict with others regarding TEM and TREG. We found no differences in TEM absolute and percentage and TREG absolute counts although others reported higher TEM absolute counts and percentage [6], and higher TREG absolute counts [8]. We found these minor differences in some T-cell subsets, but our findings do support that even among ESRD patients there is a memory shift in T-cell subsets in the elderly along with an increase in regulatory T-cells population.

Studies of B-cells, in aging, describe lower absolute counts and percentage of total B lymphocytes [9, 11-13, 30], with a higher percentage of BM [10,11, 31]. However, there is no consensus regarding quantitative impact in B naive and BREG percentages that could be unchanged [13] or diminished [11]. Our findings on the B-cells do not support any conclusions of a profile shift in these lymphocyte subsets with aging. The minimal changes observed in our B-cell results may indicate the need of a bigger sample size to clearly detect any differences and clarify if aging in ESRD results in any B-cell subset changes

This study supports the following rationale: thymus atrophy is significant in elderly leading to a secondary reduction in thymic output of lymphocyte emigrants, responsible for the replenishment of naive T-cell pool [8, 32, 33] and expansion of peripheral T- cells to maintain counts [8, 32, 34, 35]. The net result is an increase in T- cells with memory phenotype. This phenomenon is still relevant in aging even if ESRD is overlaid.

Our data showed that ESRD was also associated with global reduction in the absolute number of total lymphocytes and lower absolute TCD4 and TCD8 cell counts. In TCD4 subsets we found lower absolute counts of T naive, TCM and TEMRA, with higher percentage of TEM. The lower percentage of TREG in ESRD was divergent with our aging findings. The B-cells population, in ESRD, was associated with lower absolute B-lymphocyte counts and lower absolute counts of all B-cell subsets.

**Table 3:** Two-way Analysis of variance for ESRD, age and Age x ESRD interaction.

Lymphocyte subsets	Absolute			Percentages		
	Age^a^	ESRD	Age x ESRD^b^	Age^a^	ESRD	Age x ESRD^b^
Total	**<0.001**	**<0.001**	0.2	0.18	0.46	0.06
T	**<0.001**	**<0.001**	0.64	**0.009**	0.17	0.51
TCD4^+^	**<0.001**	**<0.001**	0.95	0.66	0.50	0.15
TCD8^+^	**<0.001**	**0.001**	0.45	**0.007**	0.41	0.41
B	0.06	**<0.001**	0.38	0.65	**0.02**	0.53
T naive	**0.04**	**0.003**	0.96	0.95	0.34	0.95
T central memory	0.41	**0.001**	0.76	**0.02**	0.39	0.62
T effector memory	0.054	0.37	0.53	0.19	**0.03**	0.79
TEMRA	**<0.001**	**<0.001**	0.55	**0.02**	0.26	0.56
Regulatory T cells	0.52	**<0.001**	0.35	**0.04**	**0.02**	0.54
B naive	0.06	**<0.001**	0.69	0.12	0.11	0.64
B memory	**0.001**	**<0.001**	0.61	0.38	0.96	0.94
Regulatory B cells	0.98	**0.04**	0.79	0.07	0.32	0.94

Abbreviations: ESRD - end-stage renal disease, TEMRA - T effector memory with RA reexpression. P values are presented. P<0.05 is significant

a Age categories of adults (18-45 years) and elderly (>60 years) were used

b Interaction term for age and ESRD in the statistical model

Our findings of ESRD are in agreement with others’, describing lower absolute total lymphocytes [16-18, 20, 36, 37], T lymphocytes [15, 36, 37] and TCD4 [15-17, 36, 37], with no changes in the relative percentage of T lymphocytes [36, 37], TCD4 [16, 36] or TCD8 [17, 36, 37], but lower T naive absolute count [17, 37], lower absolute TCM lymphocyte counts [17, 37] and higher percentage of TEM lymphocytes [18]. However our data is discordant of others who reported unchanged absolute TCD8 [17, 36, 37], lower T naive percentage [16, 18], higher percentage of TCM [18]. We have also found lower TREG percentage in ESRD which is compatible with a more pro-inflammatory profile described in uremic patients. However this finding contrast with others describing unchanged [16] or lower [15] absolute TREG counts without changes in TREG percentage [15, 16, 20, 38, 39].

Our data on B-cells in ESRD patients are in agreement with others who also describe lower B lymphocyte absolute counts [15, 36, 37] and percentage [36] with unchanged B naive and BREG percentages [20]. We diverge from others in the BM lymphocytes findings. We saw no changes in BM percentage, although others describe a lower percentage [20].

The reasons for these discrepant results may be related to different composition of the ESRD populations. The mean age difference between our elderly and younger ESRD adults were 30 years (35 vs 65y) while others present median ages in the 4^th^ and 5^th^ decades [17, 18, 20, 36, 37, 39]. Various studies also included ESRD not always on dialysis [16, 18, 20, 36, 37] and these patients not necessarily were eligible for kidney transplantation [16-18, 20, 36, 37]. Our patients were a mixture of elderly and young adults, all on dialysis for a mean of 3 years and candidates to renal transplantation while patients on dialysis in other studies presented a wide range of time on replacement therapy [16, 36].

The present research supports the idea of premature senescence in T-cell compartment among ESRD individuals with a shift toward a memory profile. CKD leads to premature decline in thymic function [40], increase in memory T-cell with terminal differentiation [40, 41] and higher apoptosis occurrence of T naive cells [40]. Consequently, lymphocyte subset changes resembling aging, with premature immunological senescence [40, 41] affecting the T compartment. B-cells seem to present a more prominent absolute count reduction without any significant profile shift. Among elderly individuals aging still have expressive effects.

Despite convergent findings with other reports on CKD and aging effects, our study analyzed, simultaneously, both effects in a number of subsets beyond the usual studies scope. Our greater novelty is the analysis of senescense and ESRD co-occurrence that resulted in greater reduction in all lymphocyte subsets, without synergic effects and little percentage changes, except for higher BREG percentage.

How could these lymphocyte changes be translated into clinical practice of renal transplantation in ESRD elderly patients? It should be pointed that higher BREG and TREG counts correlates with lower acute rejection rates [25, 42], operational tolerance [27], and higher glomerular filtration rates [25]. Also, BREG showed a protective role in kidney transplantation [26]. These findings have implication in selecting immune-suppressive drugs for the elderly recipients. Thymoglobulin, *in vivo*, results in reduction of TREG absolute counts [43] with percentage elevation [44, 45]. Calcineurin inhibitors (CNI) prevent TREG induction *in vitro* [46] in a dose dependent fashion [47], while mTOR inhibitors (mTORi) account for peripheral TREG generation *in vitro* [48] and TREG expansion in clinical practice [49]. Information about the effects of immunosuppressive drugs on BREGs is still scarce. CNIs [50, 51] and mTORi [51] seem to result in a BREG decrease, while belatacept seems to favor BREG subpopulation expansion [52].

Besides, these elderly patients may need lower IS exposure and doses [4, 5] to avoid both rejection and infection. Lower immunosuppression with relative regulatory sparing could take advantage of the elderly lymphocyte profile maintaining lower acute rejection rates and maybe lowering infection rates. The above rational is in agreement with the selection of low tacrolimus/mTORi immunosuppression in our ongoing clinical study of renal transplantation in the elderly [28].

Despite the relatively small sample sizes that could have influenced statistical discrimination of differences, our samples are similar to other studies with alike subsets evaluation [7, 8, 16, 17, 19, 24]. For final effect of each condition, we only considered significant effects that occurred simultaneously in both condition-grouped analysis. In ESRD analysis the grouped comparisons (HAd vs. EnAd and HEd vs. EnEld) presented opposite age discrepancies, consequently the simultaneously observed differences strengthen the attributed ESRD effects. In aging analysis the grouped comparisons (HAd vs. HEd and EnAd vs. EnEld) presented the same age discrepancies, but the narrower difference of age in the ESRD patients associated with the simultaneously detected differences in the healthy controls strengthen the attributed aging effects. Finally, the same aging and ESRD effects were also present in the two-way analysis of variance comparison. Based on these assumptions the sample sizes and healthy control groups may not be ideal, but they do not preclude our conclusions.

Although the current evolution of genomic technologies has enabled the discovery of important genomic markers that have been shown to correlate with CKD diagnosis and evolution [53], immune senescence [54] and successful aging [55], the current study did not account for these parameters.

In summary, our data show that both aging and ESRD result in decrease of absolute lymphocyte counts with memory profile shift, with divergent effects on TREGS. Immunosuppression regimens, in elderly recipients, that favor TREG and BREG preservation may improve clinical outcomes although a large clinical trial is warranted to prove this hypothesis.

## Supplementary Materials

The Supplemenantry data can be found online at: www.aginganddisease.org/EN/10.14336/AD.2018.0318


