# Supplementary material for: Aging and End Stage Renal Disease Cause A Decrease in Absolute Circulating Lymphocyte Counts with A Shift to A Memory Profile and Diverge in Treg Population
Source: Aging Dis. 2019 Feb 1;10(1):49–61. doi: 10.14336/AD.2018.0318 (PMC6345336; doi:10.14336/AD.2018.0318)
Supplement: Supplementary file 1 [file AD-10-01-49-s.pdf]

SUPPLEMENTARY DATA

Aging and End Stage Renal Disease Cause A Decrease in Absolute Circulating Lymphocyte Counts with A Shift to A Memory Profile and Diverge in Treg Population

Geraldo Rubens Ramos Freitas[1,2], Maria da Luz Fernandes[2], Fabiana Agena[2], Omar Jaluul[3], Sérgio Colenci Silva[3], Francine Brambate Carvalhinho Lemos[2], Verônica Coelho[4], Elias David-Neto[2], Nelson Zocoler Galante[2,*]

[1]Division of Nephrology, and [2]Renal Transplant Service, Hospital das Clinicas, University of Sao Paulo School of Medicine, Sao Paulo, Brazil. [3]Division of Geriatrics, Hospital das Clinicas, University of Sao Paulo School of Medicine, Sao Paulo, Brazil. [4]Laboratory of Immunology, Heart Institute, University of Sao Paulo School of Medicine. Institute for Investigation in Immunology, Sao Paulo, Brazil.

© 2018. Freitas GRR et al. Published online at http://www.aginganddisease.org/EN/10.14336/AD.2018.0318

SUPPLEMENTARY DATA

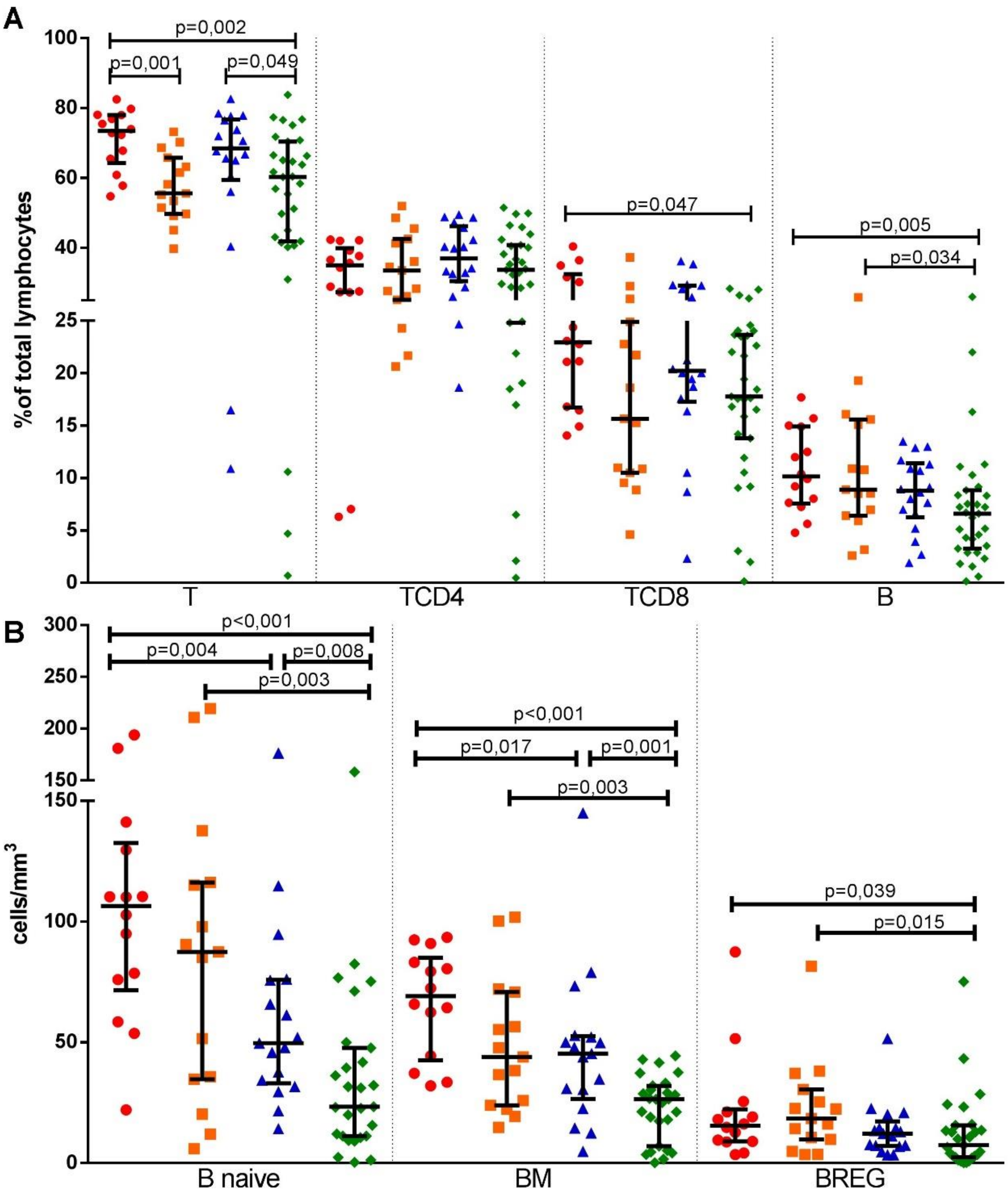

Supplementary Figure 1. Aging and end stage renal disease effects in percentages of T-, TCD4-, TCD8- and B- cells (A) and on B-cells subsets absolute counts (B). Healthy adults (n=14) (●), healthy elderly (n=15) (■), end stage renal disease adult patient (n=18) (▲) and end stage renal disease elderly patients (n=31) (◆) absolute counts and percentages are shown in the same repeatedly order in each cell subset analysis. T – T lymphocyte, TCD4 – T helper, TCD8 – T cytotoxic, B – B lymphocyte, BM – B memory, BREG – regulatory B cells. Bars represent median and interquartile ranges.

© 2018. Freitas GRR et al. Published online at http://www.aginganddisease.org/EN/10.14336/AD.2018.0318

SUPPLEMENTARY DATA

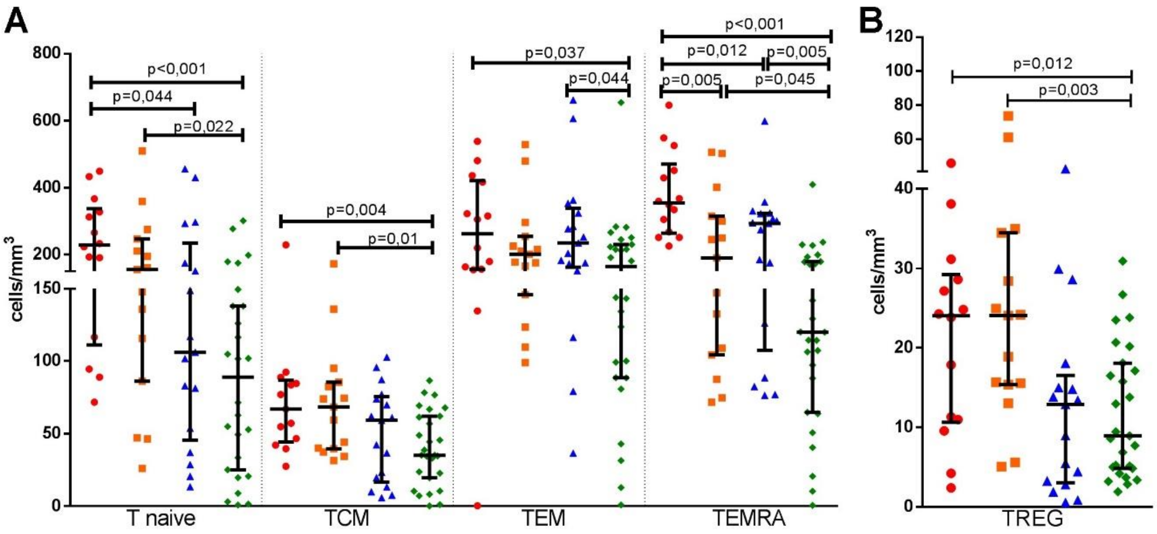

Supplementary Figure 2. Aging and end stage renal disease effects in T - cells subsets absolute counts, T naïve, TCM, TEM, TEMRA (A) and TREG (B). Healthy adults (n=14) (●), healthy elderly (n=15) (■), end stage renal disease adult patient (n=18) (▲) and end stage renal disease elderly patients (n=31) (◆) absolute counts are shown in the same repeatedly order in each cell subset analysis. TCM - T central memory, TEM - T effector memory, TEMRA - T effector memory with RA reexpression, TREG – regulatory T cells. Bars represent median and interquartile ranges.

© 2018. Freitas GRR et al. Published online at http://www.aginganddisease.org/EN/10.14336/AD.2018.0318
